# The Costs of Green Leaf Volatile-Induced Defense Priming: Temporal Diversity in Growth Responses to Mechanical Wounding and Insect Herbivory

**DOI:** 10.3390/plants8010023

**Published:** 2019-01-18

**Authors:** Jurgen Engelberth, Marie Engelberth

**Affiliations:** Department of Biology, University of Texas at San Antonio, One UTSA Circle, San Antonio, TX 78249, USA; jengelberth@gmx.net

**Keywords:** green leaf volatiles, Z-3-hexenyl acetate, plant defense, defense priming, plant growth

## Abstract

Green leaf volatiles (GLVs) have long been associated with plant defense responses against insect herbivory. Although some of their biological activities appear to directly affect the attacking herbivore, one of the major functions of GLVs seems to be the priming of these defense responses. This priming is generally considered to impose low costs on the plant should no direct attack happen. Here, we demonstrate that priming of maize seedlings with GLVs is costly for the plants as it results in significantly reduced growth. We further demonstrate that priming very selectively affects growth responses after insect elicitor treatment and mechanical wounding depending on the age and/or the developmental stage of the treated plant. The differential growth response of maize seedlings to treatment with GLVs and subsequent herbivory-related damage sheds new light on the biological activity of these important plant volatile compounds and indicates consequences that go beyond defense.

## 1. Introduction

Plants are constantly threatened by a plethora of pests and pathogens. For example, insect herbivores have managed to identify suitable host plants for oviposition and feeding through adaptation during coevolutionary processes [1]. Since many insects depend on plants as their primary food source, the survival of plants depends strongly on their ability to have a strong and also variable response to reduce damage. Consequently, the defensive responses of plants to insect herbivore attacks are regulated by a complex chemical signature that defines the outcome of this interaction. Although mechanical damage (MW) of the plant is an essential part of this interaction, insect herbivores also produce chemicals in their saliva (insect elicitors or IE) [2,3,4,5,6,7], which are sensed by the plant and contribute significantly to the regulation of the plant’s defensive reactions. This mainly occurs through the activation of the octadecanoid signaling pathway, with jasmonic acid (JA) being the major active product of this pathway [8,9,10,11,12,13,14,15]. JA subsequently activates the production of proteinase inhibitors and toxic secondary metabolites as well as the release of herbivore-induced plant volatiles (HIPV) [1,8,9,16,17]. These HIPV, which mainly consist of products of the shikimic acid pathway, terpenes and fatty acid-derived products, such as GLVs [7,8], have been shown to be a very effective countermeasure by repelling further infestation [18] and attracting predators and parasites of the attacking herbivore [19,20,21,22]. A class of very potent elicitors from insect saliva was identified as amino acid-conjugates of fatty acids, such as volicitin (17-hydroxy-N-linolenoyl-glutamine) or N-linolenoyl-glutamine [3,4,8], both of which have been shown to mimic insect herbivore damage when applied exogenously to wounded sites in maize seedlings [8].

GLVs represent a major group of HIPV and are emitted in significant quantities during insect herbivory [7]. The biosynthetic pathway for the production of GLVs is well understood [23,24,25,26,27]. GLVs are fatty acid-derived products formed from linolenic acid and linoleic acid, which serve as substrates for a pathway-specific 13-lipoxygenase (for corn LOX10 (21)). The resulting 13-hydroperoxy C_18_ fatty acid is subsequently cleaved by the enzyme hydroperoxide lyase (HPL), which produces *Z*-3-hexenal (Z-3-HAL; from 18:3 fatty acids) or hexanal (from 18:2 fatty acids) as well as 12-oxo-(*Z*)-9-decenoic acid. Further processing of *Z*-3-hexenal by alcohol dehydrogenase, acetylation and isomerization leads to the production of the remaining C_6_-components,, such as *Z*-3-hexenol (Z-3-HOL), *Z*-3-hexenyl acetate (Z-3-HAC) and the respective *E*-2-enantiomers. Although Z-3-HAL is synthesized by damaged tissue, Z-3-HOL and Z-3-HAC require intact cells for their biosynthesis [27]. GLVs are almost immediately released locally after wounding [23] but can also be produced and released systemically in response to herbivore damage [28]. 

Although GLVs may also play important roles in the immediate response to wounding, their activity as volatile defense signals between plants have become a major focus of research in the last decade. Engelberth et al. (2004) found that maize seedlings exposed to GLVs rapidly accumulate JA and emit small amounts of HIPV [29]. More importantly, maize seedlings exposed overnight to GLVs from neighboring plants produced significantly more JA and HIPV compared to their controls when treated with IE. This was the first report on priming against insect herbivory signaled by GLVs and it was demonstrated that this effect is specifically linked to defense responses as the responses to MW alone were not affected. This specific priming response driven by GLVs has since been confirmed for several other plant species, including lima beans, wild tobacco, poplar and *Arabidopsis* [30,31,32,33,34,35]. In maize, GLVs were further shown to induce many genes that are typically induced by insect herbivory and JA [16]. These significantly upregulated genes included many with proposed functions in signaling as well as those more directly involved in defense, such as proteinase inhibitors and those related to HIPV biosynthesis. This suggests that GLVs may also provide more direct protection against insect herbivory. However, GLVs are still mostly considered to be priming agents against insect herbivory. Priming of defenses is a unique response, in which the priming agent causes an enhanced or accelerated response when actually challenged by pests or pathogens [36]. Although it is unknown as to precisely how priming regulates these subsequent responses, it appears to work through one or more of the commonly-studied defense signaling pathways (salicylic acid-, JA- or ethylene-mediated). For example, in maize, GLVs not only induced a transient accumulation of JA within minutes but also primed JA-dependent responses one day later [29,37].

Since GLVs provide enhanced protection against insect herbivory, it was hypothesized that the activation of defense responses by these compounds requires a significant investment of resources, both at earlier time points, when mainly direct defenses are activated and at later time points during primed responses, which for example results in a significant increase in HIPV release in maize seedlings. Consequently, both of these investments should cause a reduction of growth and/or yield. However, to date, little is known about the costs of priming by GLVs and its effects on growth and development. To gain further insights into this matter, we started a comprehensive analysis of growth responses to Z-3-HAC as our model GLVs. We selected Z-3-HAC because intact plant cells, including those of maize plants, rapidly transform the precursors Z-3-HAL and Z-3-HOL into Z-3-HAC in a NADPH-dependent reaction [27]. Since intact plants were used for all experiments, it would be impossible to determine which of these compounds might be the active one. Furthermore, we tested Z-3-HAC-primed plants for their growth responses to IE and MW. We also used maize plants of different ages and developmental stages to test whether or not age and/or developmental status influence the response to Z-3-HAC and subsequent treatment with IE or MW. GLVs have been described to mainly affect IE responses in maize. This resulted in significantly increased JA production and HIPV release. In contrast, GLVs had no effects on MW responses [29]. We therefore investigated how a single exposure (priming) to Z-3-HAC may affect subsequent growth responses in IE- and MW-treated maize seedlings. As a consequence of the specific priming effect that GLVs have on responses to IE [29], but not to MW alone, we expected to find a differential growth response between those two treatment groups. The results show that maize seedlings respond differentially to these treatments with altered growth responses, demonstrating for the first time that (i) exposure to GLVs is costly, resulting in significantly reduced growth, and that (ii) primed responses to IE and MW result in differential growth responses, which depend on the developmental state of the plants.

## 2. Results and Discussion

### 2.1. Response to Treatment with Z-3-HAC

**Figure 1:**
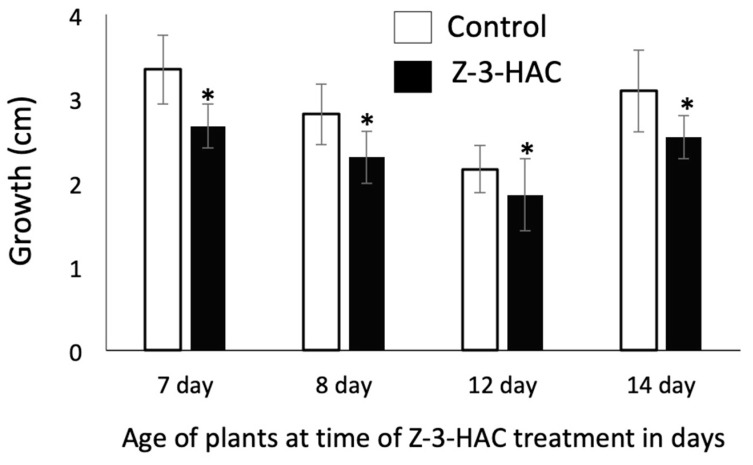
The effect of Z-3-hexenyl acetate (Z-3-HAC) exposure on growth in maize (*Zea mays*) seedlings (*n* ≥ 15) at different ages/developmental stages. Seedlings were 7-, 8-, 12- and 14-days old when treated with physiological concentrations of Z-3-HAC (20 μg L^−1^) overnight (15 h). The growth of the actively growing 2nd, 3rd, 4th and 5th leaf was measured. The values are means ± SE and the asterisks indicate significant differences between control- and Z-3-HAC-treated seedlings (*t*-test, *P* < 0.05).

When maize seedlings of different ages/developmental stages were exposed overnight to physiological concentrations of Z-3-HAC, a significant reduction in growth was observed. The reduced growth response to Z-3-HAC was constant throughout all leaf developmental stages with a growth reduction of 17–25% over the 15-h treatment period (Figure 1). We further observed an overall decline in growth in 7- to 12-day-old plants over this period while 13- and 14-day-old plants increased their growth again. This observation correlates with age exhaustion of resources, which are provided by the kernel up to day 12. The overall growth of the maize seedlings increases when photosynthesis becomes the main source of assimilates, which typically occurs after 12 days [38,39].

These results document the profound effect of GLVs on plant growth in early developmental stages and confirm that exposure to GLVs imposes a significant cost on the plants. These results were unsurprising since we had previously shown that exposure to these compounds activates a significant accumulation of defense-related transcripts, including those for proteinase inhibitors and HIPV in maize seedlings [16]. This immediate activation of defenses is costly and may thus explain the reduction in growth following treatment with Z-3-HAC.

### 2.2. Response to IE and MW Treatment after Priming with Z-3-HAC

**Figure 2:**
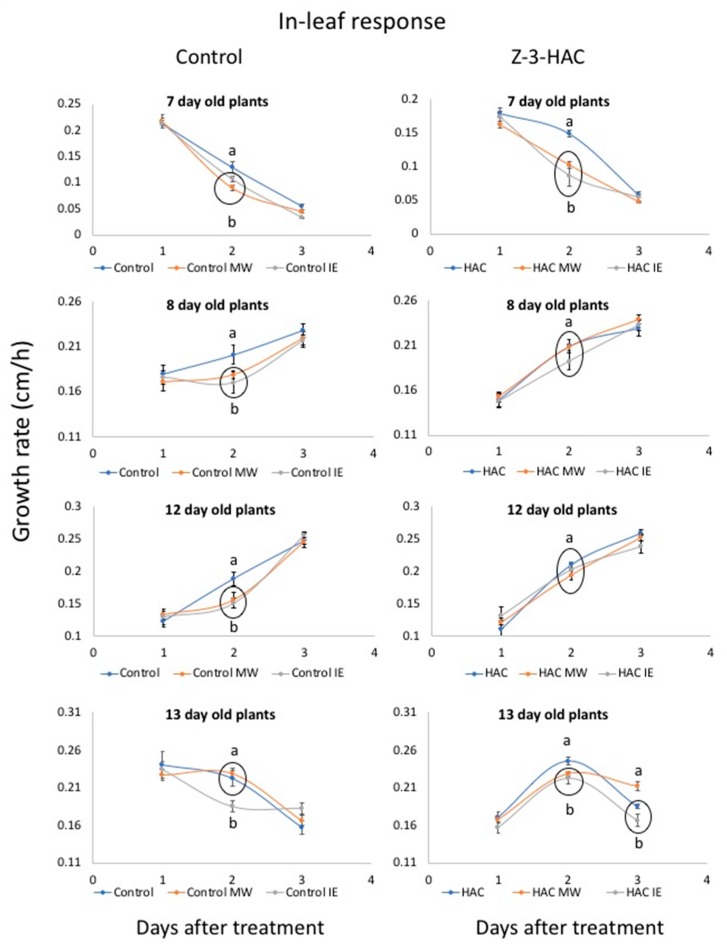
The effects of priming by (*Z*)-3-hexen-1-yl acetate (Z-3-HAC) on mechanical wounding (MW) and insect elicitor (IE) induced growth responses in maize (*Zea mays*) seedlings (expressed as growth rate in cm*h^−1^, *n* ≥ 7). Responses in control maize plants at different ages are shown in the left column and responses of Z-3-HAC-treated plants are shown in the right column. Maize seedlings were first exposed to physiological concentrations of Z-3-HAC (2 μg L^−1^ air volume) for 15 h before being an actively growing leaf was treated with MW or IE (in-leaf treatment). The growth of the treated leaf was measured for 3 days and is expressed as growth rate (cm*h^−1^). Values are means ± SE and different letters indicate significant differences between the different treatments (*ANOVA*, *P* < 0.05). Circled time points indicate no significant differences between treatments on day 2. Day 1 and Day 3 growth rates show no significant differences between treatments, with only 13-day-old Z-3-HAC-treated seedlings showing different growth rates on day 3 (circled).

We monitored the growth of treated leaves (in-leaf responses) over 3 days after exposure to Z-3-HAC (as well as in controls), with the MW and IE treatment performed on day 2 (Figure 2). We analyzed the growth response in controls and after Z-3-HAC priming of actively growing leaves of 7-, 8-, 12- and 13-day-old plants by treating the 2nd, 3th, 4th and 5th leaf in the respective maize seedling. The results show that the growth responses to Z-3-HAC priming and subsequent treatment with MW and IE were much more diverse when compared to their corresponding control plants. For in-leaf responses in control maize seedlings we found that treatment with MW and IE reduced the growth rate significantly in 7-, 8- and 12-day-old maize seedlings while 13-day-old maize seedlings did not experience significantly reduced growth rates after MW treatment (Figure 2) but responded to IE treatment with a growth reduction.

However, when maize seedlings were first treated with Z-3-HAC overnight and then with MW or IE, we found a significant reduction in growth rates only in 7- and 13-day-old seedlings while 8- and 12-day-old plants did not display any significant alterations in their growth rates.

The similar growth responses of MW- and IE-treated plants in controls as well as after Z-3-HAC priming came as a surprise considering the differential defense responses of maize seedlings to these treatments. IE treatment is very similar in its outcome to actual insect herbivory and induces much stronger defense responses than MW alone [37,40]. Furthermore, in maize leaves, these responses occur both around the damage site and in distal parts of the treated leaf while at the same time no significant activation of defense responses in basal parts of the leaf was detected [17,37,40]. In contrast, treatment with MW only induced responses in the immediate vicinity of the damage site, with no distal or basal effects. Thus, the investments in defense are significantly higher in IE-treated maize seedlings when compared to MW alone, which would suggest a much stronger reduction in growth. However, with the exception of 13-day-old control plants treated with IE, no significant differences between these treatments were found. The results in Figure 2 also clearly demonstrate that at least some signal or metabolic component during these responses affects the basal parts of the treated leaf, which is an area of active growth and results in a significant reduction of growth. However, the efficiency of this signal also seems to depend on the age/developmental stage of the plant. Furthermore, it appears that the mechanical wounding part of both, the MW- and the IE treatment, is mainly responsible for the observed growth reduction and that IE does not further reduce growth in the basal areas of the treated leaves.

**Figure 3:**
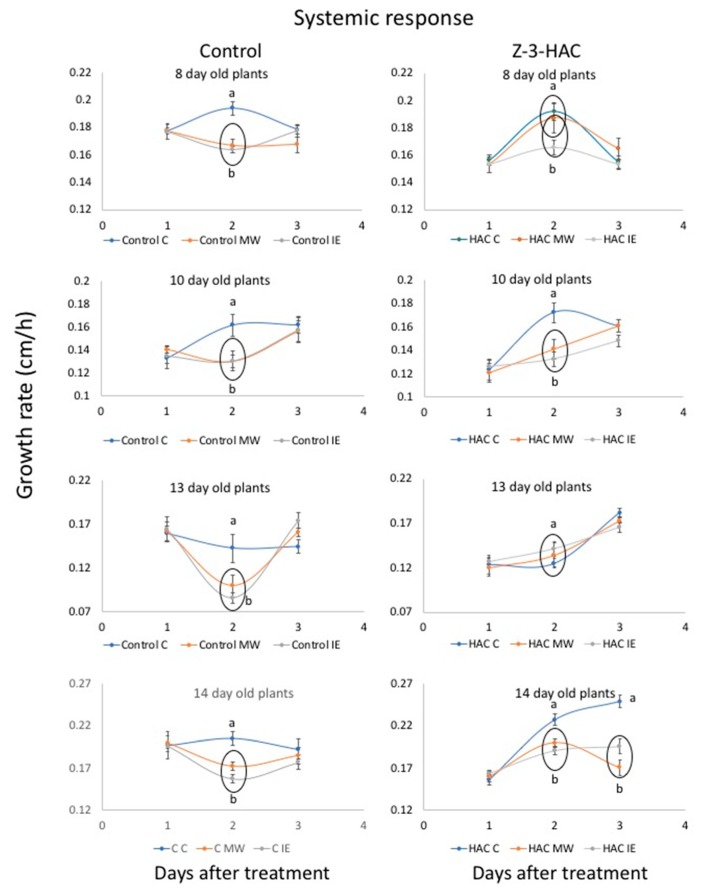
The effects of priming by (*Z*)-3-hexen-1-yl acetate (Z-3-HAC) on mechanical wounding (MW) and insect elicitor (IE) induced growth responses in systemic leaves of maize (*Zea mays*) seedlings (expressed as growth rate in cm*h^−1^, *n* ≥ 7). Responses in control maize plants at different ages are shown in the left column and responses of Z-3-HAC-treated plants are shown in the right column. Maize seedlings were first exposed to physiological concentrations of Z-3-HAC (2 μg L^−1^ air volume) for 15 h before the MW or IE treatment was applied to an actively growing leaf (7- and 12-day-old seedlings) or a fully developed leaf (8- and 14-day-old seedlings). The growth of the next (systemic) leaf was measured for 3 days and is expressed as growth rate (cm*h^−1^). Values are means ± SE and different letters indicate significant differences between the different treatments (*ANOVA*, *P* < 0.05). Circled time points indicate no significant differences between treatments. Day 1 and Day 3 growth rates show no significant differences between treatments, with only 13-day-old Z-3-HAC-treated seedlings showing different growth rates on day 3.

The previous experiment confirmed that both MW and IE treatments produced a basal signal in at least some ages/developmental stages, with a previous exposure to Z-3-HAC further modulating these responses. Thus, we also analyzed the effects of these treatments on systemic leaves. As described above for the in-leaf treatment group, we found that control plants at all ages/developmental stages showed a significantly reduced growth rate after treatment with MW and IE in the systemic leaf (Figure 3). Furthermore, both IE and MW caused the same reduction in growth rate. However, in Z-3-HAC-treated maize seedlings, the effects of MW and IE on the systemic response again differed greatly. Although IE treatment caused a significant reduction in the growth rate of 8-day-old seedlings compared to its control, MW-treated plants showed a growth rate that was similar to control plants. For 10-day-old maize seedlings, we found that both MW- and IE-treated plants had significantly reduced growth rates. In contrast, 13-day-old seedlings showed no difference in growth rate between MW, IE and control plants. However, the growth rates of MW- and IE-treated 14-day-old maize seedlings were again significantly lower than those of the controls and even stayed lower for two days after this treatment. Although these results again demonstrate an apparent response from a site located basally from the MW- and IE treatment site that resulted in systemic growth alteration, they also demonstrate a modulating effect of priming with Z-3-HAC on growth in systemic leaves.

**Figure 4:**
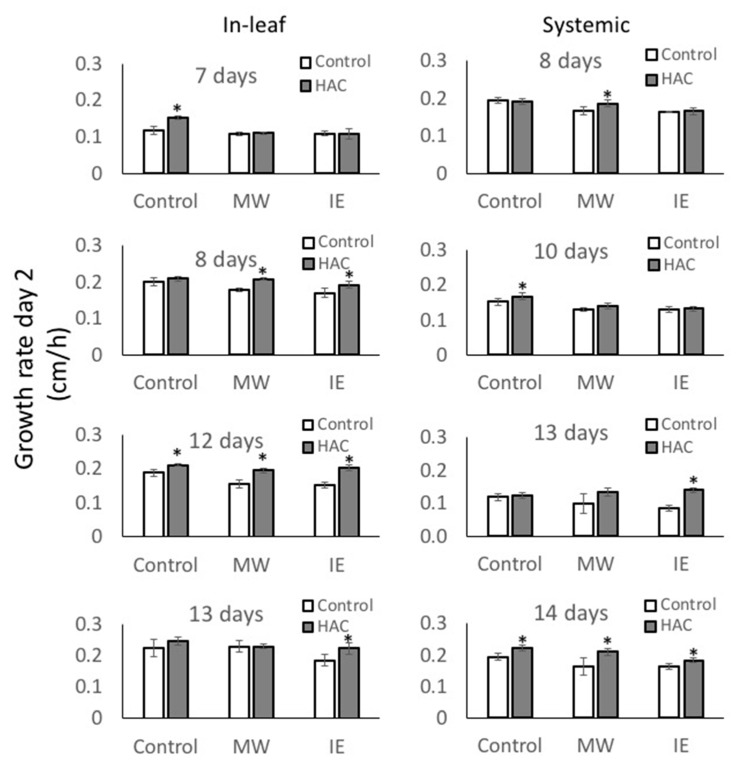
The growth response of (*Z*)-3-hexen-1-yl acetate-treated (Z-3-HAC) and untreated maize (*Zea mays*) seedlings (*n* ≥ 7) after MW and IE treatment on day 2. Seedlings were first exposed to physiological concentrations of Z-3-HAC (2 μg L^−1^ air volume) for 16 h before being treated with MW or IE as described above. Growth rate (cm*h^−1^) is shown for day 2 and is calculated as cm h^−1^. Values are means ± SE and asterisks indicate significant differences between treated and untreated seedlings (*t* test, *P* < 0.05).

Although exposure to Z-3-HAC caused a decrease in growth rate, subsequent treatment with MW or IE surprisingly did not further reduce growth in maize seedlings. When comparing the growth rates on day 2 after direct MW and IE treatments, Z-3-HAC pre-treated maize seedlings showed similar or even increased growth rates after MW and IE treatment when compared to their respective non-Z-3-HAC controls (Figure 4). This distinct growth rate behavior is surprising when considering that Z-3-HAC-primed maize seedlings subsequently challenged with IE produce nearly twice as much JA and HIPV when compared to only IE-treated controls [29]. The increased investment in defense caused by priming is not reflected in the observed growth rates in either the treated leaf or systemic leaf. Furthermore, growth responses to treatments with MW alone were also positively affected by a previous exposure to Z-3-HAC in several treatment groups. In fact, it is surprising that IE treatment generally does not cause a stronger reduction in growth than MW does with only the 13-day-old plants with the treated leaf deviating from this trend. This suggests that basal signaling from the damage site after MW and IE treatments does not depend on the abundance of elicitors but instead is mainly caused by the damage alone. Only minor attenuations in growth rates were observed after IE treatment.

One factor may be the sink–source relationship in the growing maize seedling. Older leaves generally support younger leaves with assimilates. Therefore, one might argue that by damaging the vasculature system, this transport may have become interrupted and thus may lead to reduced growth in the expanding part of the treated leaf or in the systemic leaves. However, considering the responses of 12- to 14-day-old seedlings after previous exposure to Z-3-HAC, it appears as if those leaves, although similarly damaged, respond with normal growth rates to MW and IE treatments. Furthermore, Z-3-HAC-treated control plants often show an increased growth rate when compared to their controls. For example, 7- and 12-day-old in-leaf treated maize seedlings as well as 10- and 14-day-old systemic leaves showed a significantly increased growth rate after Z-3-HAC treatment. Therefore, other yet to be identified alterations in metabolism might explain these rather normal or sometimes even increased growth rates in maize seedlings after exposure to GLVs.

**Figure 5:**
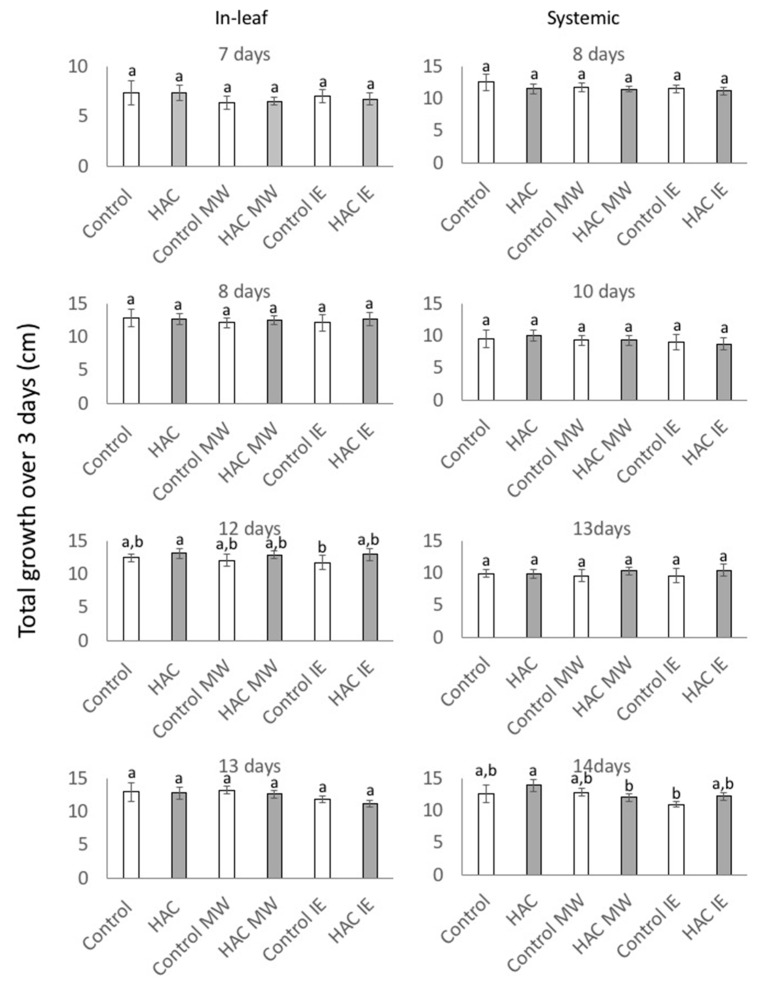
The growth response of (*Z*)-3-hexen-1-yl acetate (Z-3-HAC)-treated and untreated maize (*Zea mays*) seedlings (*n* ≥ 7) after MW and IE treatment over 3 days. Seedlings were first exposed to physiological concentrations of Z-3-HAC (2 μg L^−1^ air volume) for 16 h and then treated with MW or IE as described above. Total growth (in cm) is shown. Values are means ± SE and different letters indicate significant differences between treated and untreated seedlings (*ANOVA*, *P* < 0.05).

However, by analyzing the overall growth affected by these treatments over a period of three days, we found very few effects of either treatment on growth (Figure 5). Significant differences were only found between 7-day-old in-leaf treated Z-3-HAC Control and Control IE plants (Tukey’s HSD test, q = 4.337; p = 0.0452) and between 14 day-old Z-3-HAC and Z-3-HAC MW and Control IE plants (q = 4.502, p = 0.03 and q = 6.396, p = 0.001, respectively). Overall, induced differences in plant growth rates appear to level off after an extended period, suggesting that neither a single stimulus nor a combination of Z-3-HAC and subsequent MW or IE treatment instigates a long-lasting effect on the growth of maize seedlings. This is surprising since all three stimuli by themselves caused a significant reduction in growth in the immediate aftermath of their application. One can only conclude that mechanisms must be in place that help to alleviate those consequences. However, these mechanisms are yet to be discovered.

To summarize, we found that treatment of maize seedlings with Z-3-HAC as our model GLV caused a significant growth reduction over the first 16 hours of treatment. However, Z-3-HAC-treated maize seedlings increased their growth rate on day 2, which resulted in similar or even slightly enhanced overall growth compared to control plants. MW and IE treatments also reduced growth in treated leaves as well as systemically. Priming with Z-3-HAC was found to modulate growth responses depending on the developmental stage and/or age of the plant. However, over a period of three days, the overall growth was found to be very similar between all of these treatments, suggesting that maize seedlings have developed physiological mechanisms to compensate for losses caused by single treatments as described herein. We are currently investigating potential mechanisms that may allow maize seedlings as well as possibly other plants to alleviate the costs of metabolic investments in defenses, including those incurred by priming.

## 3. Materials and Methods

### 3.1. Chemicals

(*Z*)-3-hexen-1-yl acetate (Z-3-HAC) was purchased from Bedoukian (Bedoukian Research, Danbury, CT, USA). N-linolenoyl-glutamine was generously provided by Dr. Eric Schmelz (USDA, ARS, CMAVE, Gainesville, FL, USA) and dissolved in distilled water. All solvents used were of analytical grade.

### 3.2. Plant Material

Maize (*Zea mays* var. Kandy King) seeds (J. W. Jung Seed Co., Randolf, WI, USA) were grown in ferti-lome Ultimate Potting Mix in a growth chamber under a 12-h photoperiod at 26 °C with 60% relative humidity. Light intensity was set to ca. 150 μmol m^2^ s^−1^. For all experiments, we used 7, 8, 10, 12, 13 and 14-day-old seedlings at the V_1_, V_2_ and V_3_ stage depending on the requirements of the experiment.

### 3.3. Plant Treatments

Previously, we have shown that treatment of maize seedlings with GLVs induced a significant upregulation of defense genes, JA accumulation and HIPV release [16,17,29,37]. Since investment in these defenses should be costly, significant growth reduction in maize seedlings is expected. Furthermore, GLVs specifically increased defense responses induced by IE while responses to MW alone were not affected. It was therefore expected that effects on growth may also only be observed after treatments with IE. Furthermore, while IE mostly activates defense responses in the distal parts of leaves, little is known about the potential systemic effects of this treatment. Therefore, three independent studies were performed to analyze the effects of Z-3-HAC on the growth of leaves in maize seedlings. First, we analyzed the direct effect of Z-3-HAC on the growth of leaves in maize seedlings. Secondly, we analyzed the effects of Z-3-HAC treatments and subsequent treatments with MW and IE on in-leaf growth responses to test for direct effects of those treatments on the treated leaf. Thirdly, we analyzed the effects of Z-3-HAC treatment and subsequent treatments with MW and IE on systemic growth responses.

#### 3.3.1. Effects of Z-3-HAC on Leaf Growth

To analyze the effects of Z-3-HAC on growth, we selected 7-, 8-, 12- and 14-day-old maize seedlings for our study. Fifteen plants were used for each treatment group. Experiments started at 7 p.m. at the onset of darkness. The leaf height of an actively growing leaf was measured (from soil surface to leaf tip) before plants were exposed to Z-3-HAC. The maize seedlings were then placed in a 10-L glass cylinder and 30 µL of Z-3-HAC at a concentration of 1 µg/mL diluted in dichloromethane (DCM) was added to each glass cylinder. Control seedlings only received 20 µL of DCM but were otherwise treated similarly. The plants were then kept in their regular day/night cycle for 15 h. The leaf height was again measured and growth over the observed period was determined.

#### 3.3.2. Within-Leaf Responses

To study the effects of Z-3-HAC on mechanical wounding (MW)- and insect elicitor (IE)-related growth responses in a treated leaf, we selected 7-, 8-, 12- and 13-day-old plants. In those plants, the treated leaf (2nd, 3rd, 4th and 5th, respectively) was clearly visible and actively growing. This was necessary to ensure that the leaf was exposed to Z-3-HAC and allows for the treatment with MW and IE. Furthermore, the leaf should continue to grow for at least three more days after the onset of the experiment. Experiments started at 7 p.m. at the onset of darkness. The leaf height of an actively growing leaf was measured (from soil surface to leaf tip) before plants were exposed to Z-3-HAC. The maize seedlings were then placed in a 10-L glass cylinder and 30 µL of Z-3-HAC at a concentration of 1 µg/mL diluted in dichloromethane (DCM) was added to each glass cylinder. Control seedlings only received 20 µL of DCM but were otherwise treated similarly. The plants were then kept in their regular day/night cycle for 15 h. At 10 a.m. on the next day, the plants were removed from the glass cylinders and leaf height was again measured. Each treatment group (Z-3-HAC and Control) was then divided into three subgroups, namely control, MW treatment and IE treatment. For the analysis of in-leaf growth responses to MW and IE treatments, a small section on the upper part of the growing leaf was scratched with a razor blade (MW) or 10 µL of diluted insect elicitor (N-linolenoyl-glutamine in water; 100 pmol/μl in distilled water) was added to the damage site (IE). Since growth occurs mainly in the basal part of a maize leaf, this ensured that the actively growing area was not directly damaged and that growth responses were mainly caused by factors produced at the damage site located further distal from this area rather than through direct damage to growing cells. Leaf height was measured for two consecutive days. To allow for better comparison, growth rates (cm/h) were calculated since plants were not measured at equal intervals over the treatment period and results were compared among the different treatment groups. At least seven maize seedlings were used for each treatment.

#### 3.3.3. Systemic Responses

To analyze the effects of Z-3-HAC exposure on MW and IE induced systemic growth responses we selected 8-, 10-, 13- and 14-day-old plants. In 8- and 13-day-old plants, the MW- and IE-treated leaves (2nd and 3rd leaf, respectively) were still growing while in 10- and 14-day-old plants, those treated leaves were already fully developed at the time of IE and MW treatment. At the time of treatment, the systemic leaves (3rd and 4th leaf, respectively) were still actively growing. The leaf height of these actively growing systemic leaves was first measured (from soil surface to leaf tip) before plants were exposed to Z-3-HAC as described above. At 10 am on the next day, the plants were removed from the glass cylinders and leaf height was again measured. Each treatment group (Z-3-HAC and Control) was then divided into three subgroups (control, MW, IE). Plants were treated with MW and IE as described above and the leaf height of the untreated systemic leaves was measured for two more consecutive days. To allow for better comparison, growth was measured and growth rates (cm/h) were calculated since plants were not measured at equal intervals over the treatment period and results were compared among the different treatment groups. At least seven maize seedlings were used for each treatment.

### 3.4. Statistical Analysis

Data are presented as means ± standard errors (SE) of at least seven biological replicates per treatment, with growth being the dependent variable and Z-3-HAc-, MW- and IE treatments being independent variables. Pairwise comparisons, e.g., Z-3-HAc versus Control (Figure 1, Figure 4), were analyzed for significance with *t*-tests (*p* < 0.05). ANOVA was performed for multiple comparisons. Since growth rates varied significantly over the observed growth periods, multiple comparisons were only performed for results on a particular day during the treatment period within a treatment group (Control or Z-3-HAc) or when responses over the entire treatment period were analyzed. Significant treatment effects were investigated when the main ANOVA effects were significant (*p* ≤ 0.05). Tukey’s HSD *p*-values for multiple comparisons were used to determine significant differences between the respective control and treatments (*p* ≤ 0.05). Before statistical analysis, all data were subjected to square root transformation to compensate for elevated variation associated with larger mean values.
